# A Rare Case of Diffuse Large B-cell Lymphoma in the Breast: Diagnostic Insights and Treatment Approaches

**DOI:** 10.7759/cureus.73069

**Published:** 2024-11-05

**Authors:** Fadila Kouhen, Othmane Bensalah, Hafsa Chahdi, Adil Elghanmi, Nouama Bouanani

**Affiliations:** 1 Radiotherapy, International University Sheikh Khalifa Hospital, Laboratory of Neurooncology, Oncogenetic, and Personalized Medicine, Mohammed VI Center for Research and Innovation, Mohammed VI University of Sciences and Health (UM6SS), Casablanca, MAR; 2 Pathology, Mohamed V Military Instruction Hospital, Rabat, MAR; 3 Gynecology and Obstetrics, Faculty of Medicine, Mohammed VI University of Sciences and Health (UM6SS), Casablanca, MAR; 4 Hematology, Faculty of Medicine, Mohammed VI University of Sciences and Health (UM6SS), Casablanca, MAR

**Keywords:** breast, chemotherapy, complete remission, diffuse large b-cell lymphoma, radiotherapy

## Abstract

Diffuse large B-cell lymphoma (DLBCL) is a rare and aggressive form of non-Hodgkin lymphoma (NHL) that can occur in the breast. This case report details the case of a 48-year-old postmenopausal woman diagnosed with primary breast DLBCL after presenting with a painless, rapidly growing mass. Imaging studies, including ultrasound and mammography, indicated significant breast involvement, confirmed by histopathological analysis revealing large atypical lymphoid cells. The patient underwent the rituximab plus cyclophosphamide, doxorubicin, vincristine, and prednisone (R-CHOP) chemotherapy regimen, achieving complete remission after four cycles. This case underscores the importance of considering breast lymphoma in differential diagnoses for breast masses and highlights the effectiveness of timely and appropriate treatment strategies. Ongoing follow-up is essential for monitoring potential recurrences, emphasizing the need for a multidisciplinary approach to managing this condition.

## Introduction

Diffuse large B-cell lymphoma (DLBCL) is the most common form of non-Hodgkin lymphoma (NHL), typically affecting adults, with an average age of diagnosis between 60 and 65 years. Although it is a prevalent subtype of NHL, DLBCL is rare as a primary breast tumor, representing less than 1% of all breast malignancies and an even smaller fraction among extranodal lymphomas [1].

Unlike breast cancer, which arises from the breast tissue itself, breast lymphoma originates in lymphocytes, the white blood cells of the immune system.

While its etiology remains largely unknown, there are no established risk factors, although associations with immunosuppression and autoimmune disorders have been suggested [2]. The differential diagnosis of large B-cell lymphoma encompasses a spectrum of benign and malignant breast conditions, including breast cancer, benign breast lesions such as fibroadenomas, inflammatory breast conditions like mastitis, and metastatic disease from other primary sites [3].

Clinically, breast lymphoma often presents as a painless, palpable mass in the breast, which can lead to suspicion of breast cancer. However, it's essential to distinguish between the two because their treatment approaches differ significantly. While most cases involve only one breast, bilateral involvement is exceedingly rare, occurring in approximately 11% of cases.

The diagnosis of DLBCL requires a multidisciplinary approach. Imaging techniques such as mammography, ultrasound, and MRI are used to visualize the mass and guide tissue sampling. Histopathological examination of biopsy samples is crucial for confirming the diagnosis and determining the subtype of lymphoma.

The treatment typically involves a combination of chemotherapy and radiation therapy. Chemotherapy regimens, such as R-CHOP, short for rituximab plus cyclophosphamide, doxorubicin, vincristine, and prednisone, are commonly used to target the lymphoma cells [4]. Following chemotherapy, radiation therapy may be administered to the affected breast and regional lymph nodes to eliminate any residual disease. surgery reserved for specific situations [5]. 

Despite its rarity, ongoing research into breast lymphoma's molecular biology and genetics is shedding light on its underlying mechanisms and potential targeted therapies. Clinical trials play a vital role in evaluating new treatment approaches and improving outcomes for patients with this rare disease.

The aim of this article is to report a case involving a patient diagnosed with DLBCL in the breast. By detailing this case, we seek to enhance awareness of breast lymphoma as a critical differential diagnosis for patients presenting with breast masses, ultimately contributing to improved patient outcomes through informed clinical practice.

## Case presentation

**Figure 1:**
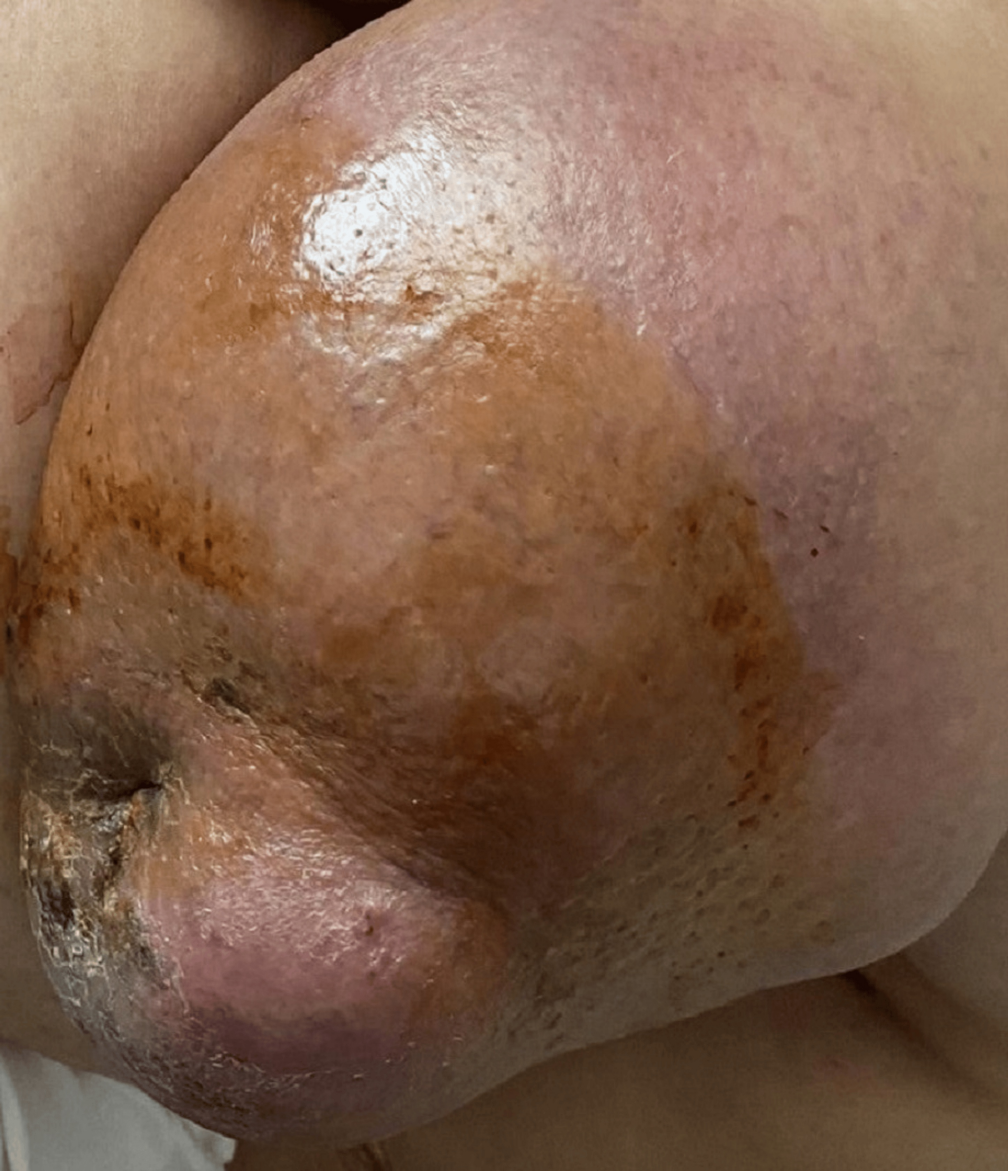
Clinical presentation of the right breast mass exhibiting inflammatory signs and nipple retraction

The clinical presentation involves a 48-year-old postmenopausal woman who reported a painless enlargement of the right breast over the course of two months. Her performance status was classified as one on the WHO scale, indicating that she was relatively active and was not bedridden. Clinical examination revealed a mass that nearly occupied the entirety of the right breast, measuring 15 cm, with associated signs of inflammation and nipple retraction (Figure 1).

There were no systemic symptoms such as fever, night sweats, or significant weight loss, and no evidence of splenomegaly, gingival hypertrophy, or mucocutaneous hemorrhagic syndrome.

Breast ultrasound identified a hypoechoic, heterogeneous, hypervascular mass with associated skin and subcutaneous thickening, alongside infracentimetric right axillary lymphadenopathy. Mammography characterized the lesion as a dense, water-density mass, relatively well-defined, measuring 116×80 mm and occupying nearly half of the right breast.

**Figure 2:**
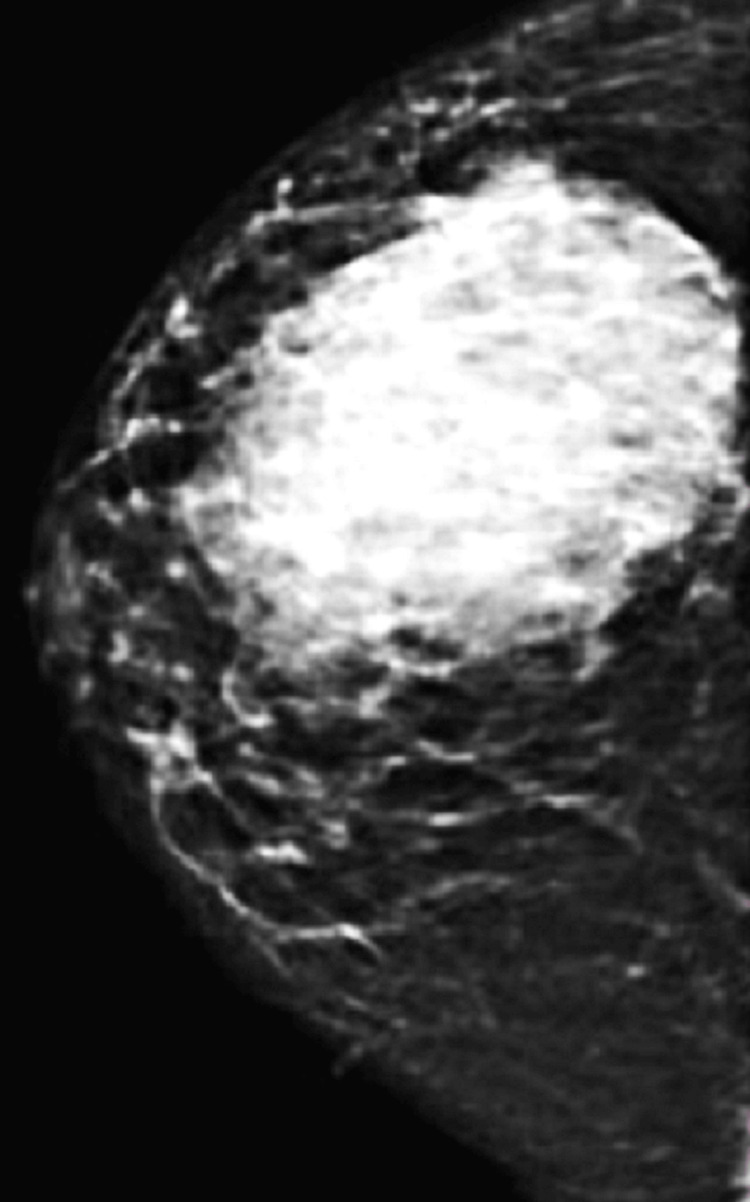
Oblique mammographic incidence demonstrating a large suspicious lesion with irregular margins

There was associated skin thickening but no suspicious microcalcifications, resulting in a Breast Imaging Reporting and Data System (BI-RADS 4) classification for both breasts (Figure 2).

**Figure 3:**
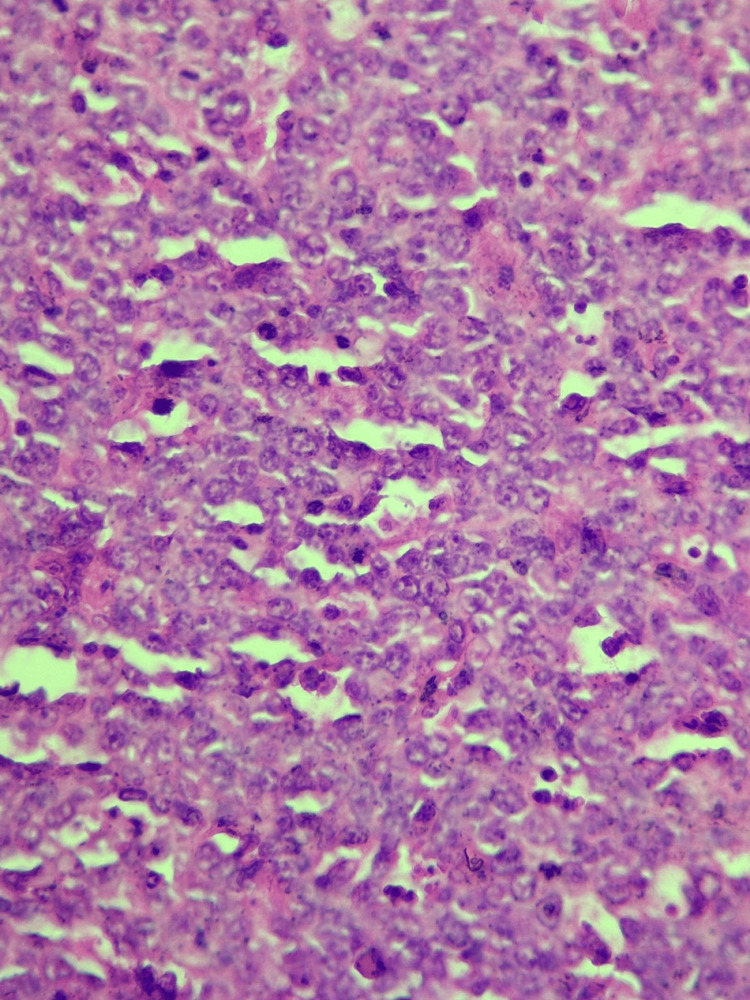
Hematoxylin and eosin staining at 40x magnification; diffuse lymphomatous tumor proliferation comprising large cells with sparse cytoplasm and hyperchromatic nuclei are noted.

Histopathological analysis from an ultrasound-guided core biopsy revealed extensive dermal infiltration by a diffuse sheet of large, poorly differentiated cells. These cells displayed hyperchromatic, often nucleolated, round to oval nuclei with scant cytoplasm, and several abnormal mitoses were observed (Figure 3).

**Figure 4:**
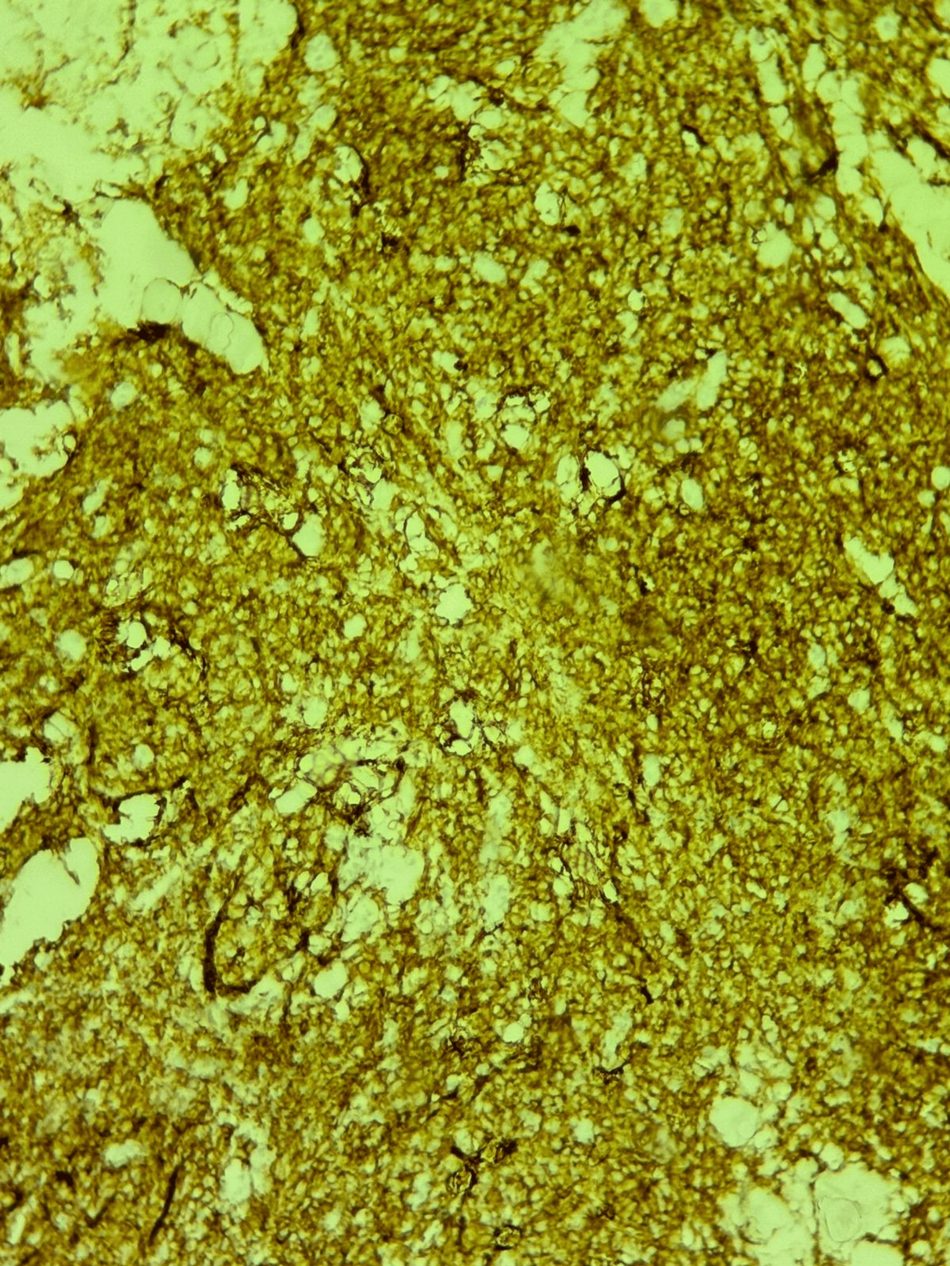
Positive CD20 staining in immunohistochemical analysis

Immunohistochemistry showed strong positivity for CD20 (Figure 4) and a Ki-67 index of 90%, while the cells were negative for cytokeratin (AE1/AE3), CD3, CD10, and CD5. These findings confirmed a diagnosis of DLBCL.

Further staging involved a negative bone marrow biopsy and lumbar puncture, indicating no evidence of hematological involvement. A thoracic-abdominal-pelvic CT scan did not reveal any additional sites of disease, suggesting localized involvement.

**Figure 5:**
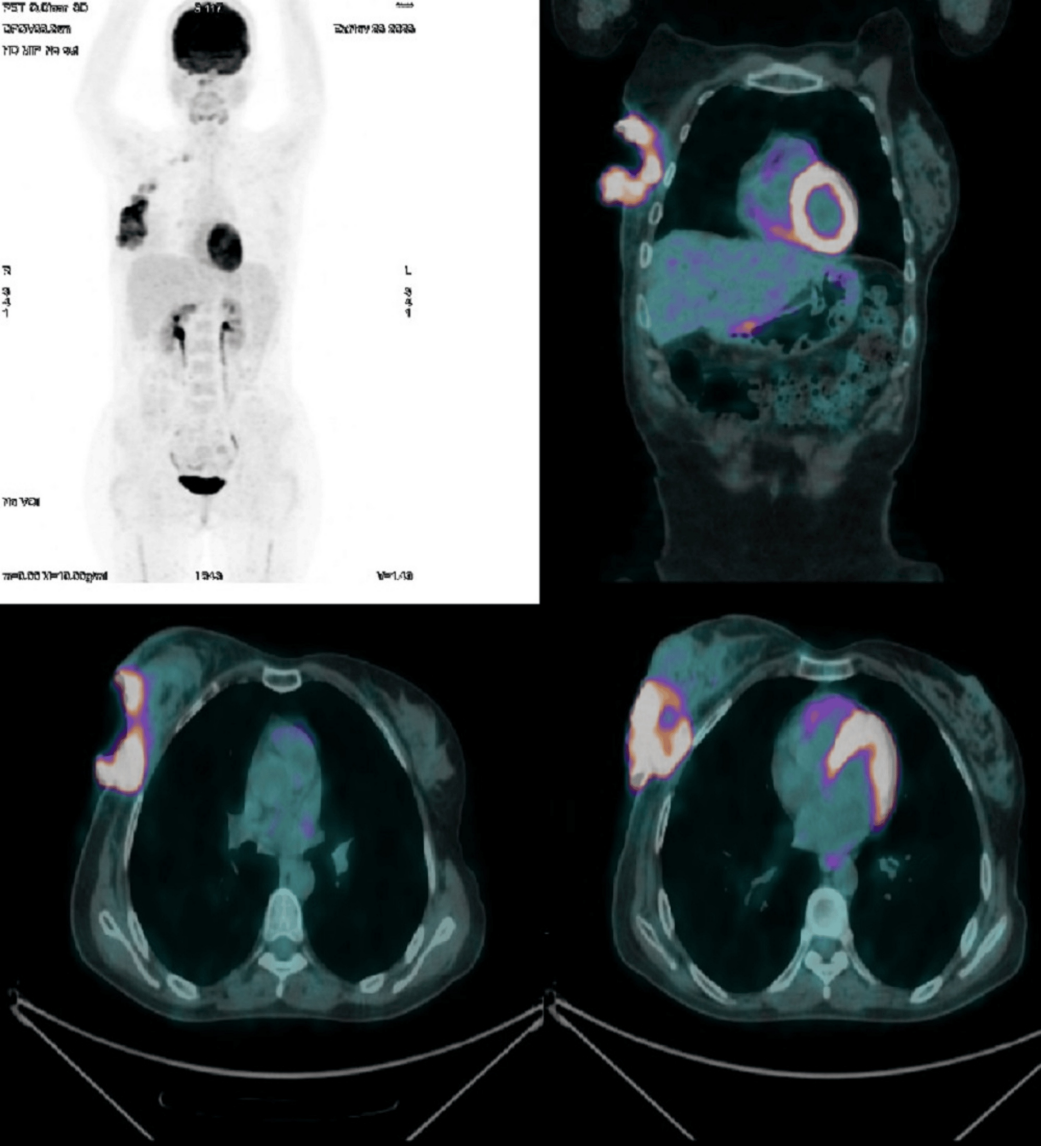
Axial and coronal reconstructions of PET scan (18F-FDG) demonstrating hypermetabolism of a large right breast mass (SUV 30) without evidence of distant adenopathy or secondary lesions. FDG: fludeoxyglucose; SUV: standardized uptake value

Subsequently, a PET/CT scan was performed, providing a comprehensive evaluation of metabolic activity in the suspected areas. The scan revealed hypermetabolism of a large right breast mass (standardized uptake value (SUV) 30) without evidence of distant adenopathy or secondary lesions (Figure 5).

Based on these imaging studies and clinical findings, the patient was staged as IE according to the Ann Arbor classification, with an International Prognostic Index (IPI) score of one and a Central Nervous System International Prognostic Index (CNS-IPI) score of two.

Laboratory investigations revealed elevated lactate dehydrogenase (LDH) levels at 1000 U/L, while complete blood count, serum electrolytes, renal function tests, and other biochemical parameters including phosphorus, calcium, uric acid, β2-microglobulin, and albumin were within normal limits.

The patient was treated with the R-CHOP regimen, which includes rituximab, cyclophosphamide, doxorubicin, vincristine, and prednisone. This treatment protocol was administered on days one and 21, for a total of eight cycles.

The infusions were conducted in an outpatient setting, with close monitoring for adverse effects such as nausea, infections, and cytopenias. Regular laboratory tests, including complete blood counts and liver function tests, were performed to assess the patient’s tolerance to therapy and to guide any necessary dose adjustments. The interval between treatment cycles was adhered to as scheduled, and no significant adverse effects were observed throughout the course of therapy.

**Figure 6:**
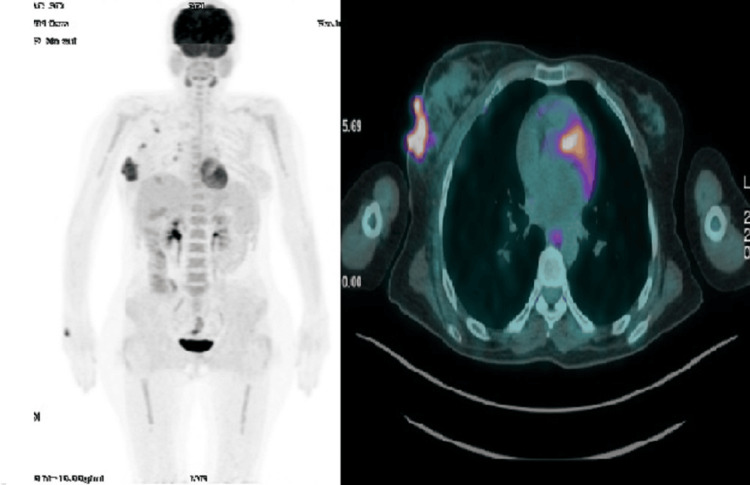
Axial and coronal reconstructions of PET scan (18F-FDG) demonstrating clear regression in size and intensity of the breast mass after the second cycle of chemotherapy FDG: fludeoxyglucose

To reduce the risk of CNS involvement, CNS prophylaxis was implemented through intrathecal chemotherapy, consisting of eight administrations of a combination of methotrexate, cytarabine, and hydrocortisone, aimed at preventing CNS relapse. The PET scan after the second cycle demonstrated an excellent therapeutic response, with a notable decrease in the size and intensity of the mass (Figure 6).

Subsequent PET imaging after four cycles confirmed a complete response.

The case was presented at the multidisciplinary tumor board, where it was determined that, given the substantial initial tumor size, the addition of radiotherapy would be beneficial, whereas surgical intervention was not indicated. The patient received a total dose of 30 Gy, administered in daily fractions of 2 Gy, with treatment sessions scheduled five days a week.

**Figure 7:**
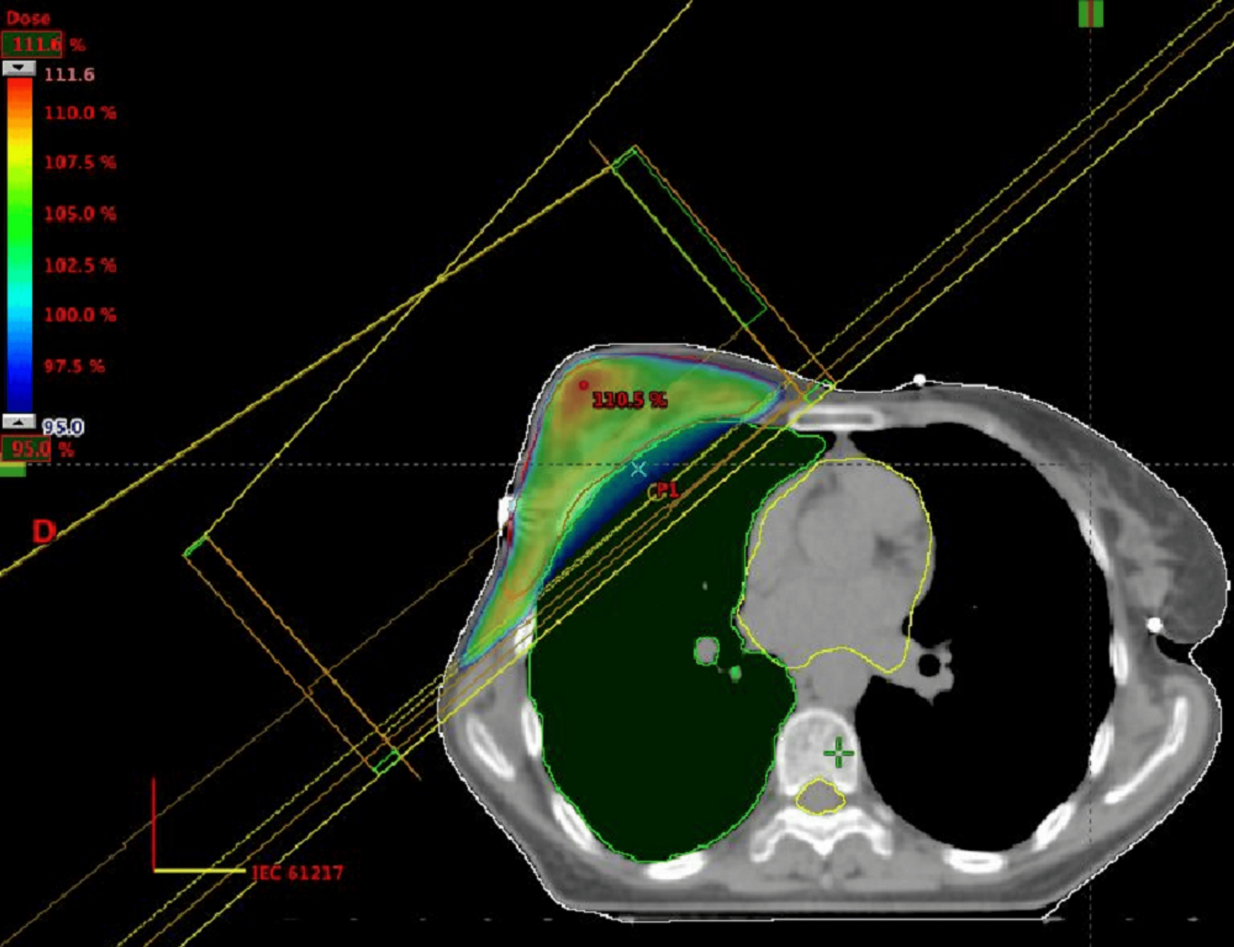
Graphical representation of radiotherapy treatment planning using the field-in-field technique

Radiotherapy was delivered using two tangential fields employing the field-in-field (FIF) technique, which allows for improved dose distribution and sparing of surrounding healthy tissues (Figure 7).

Strict dosimetric constraints were maintained to protect critical organs at risk, including the heart, right lung, spinal cord, and esophagus, ensuring minimized exposure and reduced potential for adverse effects.

Continuous follow-up was monitored for any signs of recurrence, with regular clinical assessments and imaging studies planned as part of the surveillance strategy. The patient has maintained complete remission status for two years, demonstrating a favorable response to treatment.

## Discussion

Primary breast DLBCL is a rare and aggressive extranodal NHL subtype, representing less than 0.5% of breast malignancies and around 1% of extranodal lymphomas [6]. Unlike secondary breast lymphoma, which results from systemic dissemination, primary breast DLBCL originates within the breast, often in the absence of systemic disease at the time of diagnosis.

The physiopathogenesis of DLBCL involves a complex interplay of genetic mutations, chromosomal translocations, and immune system dysfunctions that contribute to the development and progression of lymphoma [7]. Common genetic abnormalities include mutations in oncogenes such as MYC, BCL2, and BCL6, which lead to uncontrolled cell growth. With its rich lymphatic network, the breast tissue microenvironment provides a suitable niche for lymphoid cell proliferation. Chronic inflammation, such as that seen in mastitis, may further promote lymphomagenesis by creating an environment conducive to continuous cell turnover and DNA damage. Although less clear, hormonal influences and potential infectious agents like Epstein-Barr virus (EBV) might also play roles in breast lymphoma development [8]. 

It typically presents as a unilateral, rapidly growing, painless breast mass, which can be mistaken for primary breast carcinoma. The tumor may also cause skin thickening, erythema, or axillary lymphadenopathy, although constitutional "B symptoms" (fever, weight loss, night sweats) are rare in this context.

The radiographic characteristics of breast lymphoma lack distinct diagnostic indicators, often requiring a biopsy for definitive confirmation.

Mammography typically reveals breast lymphoma as a solitary mass, occasionally presenting as an asymmetry with an absence of calcifications. On ultrasound, breast lymphoma commonly appears as a hypoechoic mass with diverse features, including irregular shape, variable margins, and usually parallel orientation. MRI enhances diagnostic precision, revealing features such as lymphomatous masses appearing isointense to mildly hyperintense on T1-weighted imaging (T1WI) and hyperintense on T2-weighted imaging (T2WI). Variations in signal intensity reflect histological subtypes and the presence of necrosis. Dynamic contrast enhancement displays avid and heterogeneous enhancement patterns, often with persistent or plateau enhancement curves. Additionally, diffusion-weighted imaging (DWI) may demonstrate restricted diffusion within the mass, indicative of cellular density and higher cellularity. Magnetic resonance spectroscopy findings include elevated choline peaks and decreased lipid peaks, reflecting increased cellular turnover and reduced adipocyte content. Morphologically, breast lymphomas may present as solitary masses or diffuse infiltrative lesions, characterized by ill-defined margins and associated skin thickening or architectural distortion in infiltrative cases [9]. It's important to note that while intramammary lymph nodes may be present, they are not specific to lymphoma and can also be observed in other types of breast malignancies.

Diagnosis is usually confirmed through core needle biopsy, with histopathological analysis revealing diffuse infiltration by large, atypical lymphoid cells. These cells express B-cell markers, including CD20, CD79a, and PAX5, while immunohistochemistry (IHC) often shows positivity for BCL-6 and CD10, particularly in the germinal center B-cell-like (GCB) subtype. On the other hand, the activated B-cell-like (ABC) subtype is typically negative for CD10 and BCL-6 but positive for MUM1, MYC, BCL2, and BCL6 rearrangements, particularly double-hit or triple-hit lymphomas (with concurrent MYC and BCL2 or BCL6 translocations), are associated with poor prognosis and may require more aggressive treatment.

The criteria outlined by Wiseman and Liao in 1972 for diagnosing primary breast lymphomas (PBLs) encompass two fundamental elements [10]. Firstly, histopathological examination is pivotal, necessitating the identification of both mammary tissue and lymphomatous infiltrate in intimate proximity within an adequate pathological specimen. Secondly, the diagnosis requires the exclusion of widespread lymphoma involvement through standard staging techniques. Additionally, there should be no antecedent history of extramammary lymphoma. Nonetheless, the presence of ipsilateral axillary node involvement may be admissible if concomitantly diagnosed with breast lymphoma, suggesting regional rather than systemic dissemination. These rigorous criteria are essential for ensuring precise diagnosis and appropriate therapeutic strategies for PBLs, facilitating effective management of this rare clinical entity.

The standard diagnostic workup includes PET-CT to assess for extranodal involvement and to stage the disease, alongside bone marrow biopsy to rule out marrow infiltration.

Therapeutically, the R-CHOP regimen remains the cornerstone of treatment for primary breast DLBCL [11]. Rituximab, a monoclonal antibody against CD20, has markedly improved outcomes in patients with DLBCL. In localized disease, consolidative radiotherapy to the breast (typically 30-40 Gy) may be employed to reduce the risk of local recurrence [12]. Central nervous system prophylaxis with intrathecal methotrexate or systemic high-dose methotrexate is recommended in selected patients due to the increased risk of CNS involvement, particularly in those with high-risk features (e.g., high IPI score, elevated LDH, large tumor size, or involvement of multiple extranodal sites) [13].

Surgical procedures are reserved for diagnostic purposes, with minimally invasive biopsies serving to confirm histological diagnosis and guide subsequent therapeutic decisions. Extensive surgeries like radical mastectomy and axillary dissection offer no incremental benefit and may impose unnecessary morbidity [14]. 

Prognosis in primary breast DLBCL is influenced by several factors, including the IPI, which incorporates age, tumor stage, performance status, and LDH levels. Molecular subtype (GCB vs. ABC) and the presence of MYC and BCL2 or BCL6 rearrangements (as seen in double- or triple-hit lymphomas) also significantly affect outcomes, with double-hit lymphomas typically exhibiting more aggressive behavior and poorer survival rates. The five-year overall survival rate for primary breast DLBCL can range from 50% to 80%, depending on the stage at presentation, molecular subtype, and response to treatment [15]. 

Long-term follow-up is critical to monitor for recurrence, both locally and systemically, including the contralateral breast, and for potential late complications related to treatment. Multidisciplinary management, involving oncologists, hematologists, radiologists, and pathologists, is essential to optimize treatment strategies and improve patient outcomes in this rare but aggressive disease.

The origin of DLBCL, whether primary or secondary, also influences therapeutic outcomes. Primary breast lymphomas exhibit superior survival rates compared to their secondary counterparts, underscoring the importance of tailoring treatment strategies to disease etiology. Ongoing research endeavors, particularly in CNS prophylaxis, hold promise for further refining DLBCL management strategies and improving patient prognosis.

## Conclusions

In conclusion, this case of DLBCL highlights the critical need for clinicians to consider breast lymphoma as a differential diagnosis for breast masses. The successful treatment with the R-CHOP regimen led to complete remission, emphasizing the importance of timely diagnosis and appropriate management strategies. This case serves as a reminder of the potential for favorable outcomes when breast DLBCL is recognized and treated early, reinforcing the value of a multidisciplinary approach in improving patient care and long-term prognosis.
